# Serum erythropoietin levels in essential thrombocythemia: phenotypic and prognostic correlates

**DOI:** 10.1038/s41408-018-0157-5

**Published:** 2018-11-19

**Authors:** Natasha Szuber, Sravanthi Lavu, Mythri Mudireddy, Maura Nicolosi, Domenico Penna, Rangit R. Vallapureddy, Terra L. Lasho, Christy Finke, Curtis A. Hanson, Rhett P. Ketterling, Animesh Pardanani, Naseema Gangat, Ayalew Tefferi

**Affiliations:** 1 Division of Hematology, Department of Internal and Laboratory Medicine, Mayo Clinic, Rochester, MN USA; 2 Division of Hematopathology, Department of Internal and Laboratory Medicine, Mayo Clinic, Rochester, MN USA; 3 Division of Laboratory Genetics and Genomics, Department of Internal and Laboratory Medicine, Mayo Clinic, Rochester, MN USA

Serum erythropoietin (EPO) level below the normal reference range is widely accepted as a minor diagnostic criterion for polycythemia vera (PV) and has retained its value in the revised 2016 World Health Organization (WHO) classification scheme of myeloid neoplasms^1^. While over 80% of PV patients exhibit subnormal EPO values^2,3^, very little is known about the incidence, phenotypic connotation, or prognostic significance of serum EPO levels in essential thrombocythemia (ET). Studies on this subject remain, in fact, exceedingly rare^2,4–9^. Previous reports limited to small patient populations have suggested that subnormal EPO levels may be more prevalent than expected in ET, up to 50% in some series^4,6,7^. Furthermore, one study comprising 39 untreated ET patients revealed a heightened risk of vascular events and significantly shorter time to initiation of myelosuppressive therapy in those exhibiting subnormal EPO values^4^. These observations, albeit preliminary, raise the possibility that serum EPO may be *relevant*, perhaps even prognostically impactful in ET. Accordingly, after securing a diagnostically pure cohort of untreated strictly WHO-defined chronic phase ET patients, we documented serum EPO measurements, laboratory and clinical features, and overall, myelofibrosis-free, and polycythemia vera-free survival stratified by EPO level.

The current study was approved by the Mayo Clinic Institutional Review Board. Diagnosis of ET was in strict accordance with the 2016 WHO criteria^1^. ET patients with available EPO measurements at diagnosis or during their chronic phase (March 1994–November 2017) were considered under the following strict provisions: (i) all patients were in chronic phase with no evidence of myelofibrotic or leukemic transformation; (ii) all patients were untreated with cytoreductive agents at the time of sample collection and those with EPO levels drawn at diagnosis were also previously untreated with cytoreductive agents; (iii) all patients had hemoglobin levels above sex-adjusted values for anemia (no more than two standard deviations below the mean or >13.5 g/dL in men and >12.0 g/dL in women), thus excluding anemia as a potential confounding variable; and (iv) all patients with hemoglobin values exceeding the threshold for PV criteria had clinical and morphological parameters carefully assessed and all cases of masked PV systematically excluded. Few individuals with hemoglobin values exceeding the threshold for PV but without meeting PV criteria were found to have either transient elevations in hemoglobin or clear underlying causes of secondary polycythemia such as hypoxia, smoking, or elite athletic training, among others.

Serum erythropoietin levels were obtained using standard immunoenzymatic assay methods^10^ with reference range between 4 and 21 mIU/mL. Reasons for EPO draw were varied and included primarily diagnostic work-up or monitoring of chronic phase disease. Laboratory, clinical, and cytogenetics data corresponding to time of EPO sample collection and/or diagnosis were abstracted. Screening for driver mutation status was performed using conventional methods. Risk stratification was based on the three-tiered prognostic model international prognostic score for ET (IPSET)^11^.

Standard statistical methods were used to compare variables across EPO-stratified groups. Time-to-event curves were prepared using the Kaplan–Meier method and compared by the log-rank test with *p* values < 0.05 considered significant. The JMP® Pro 13.0.0 software package was used for all analyses (SAS Institute, Cary, NC, USA).

The laboratory and clinical characteristics of 177 informative cases of untreated WHO-defined ET stratified by subnormal versus normal/above normal EPO level are presented in Table 1. A substantial proportion of ET patients (*n* = 62, 35%) exhibited subnormal serum EPO values, while the remainder had values that were either normal (*n* = 113) or above normal (*n* = 2) (the latter two combined for analyses). Median age was 51 years (range 18–88) with 71% female preponderance. Median serum EPO concentrations were 5.2 mIU/mL (range <1–25) overall and 2.6 mIU/mL (range <1–3.9) and 7.2 mIU/mL (range 4–25) in those with subnormal versus normal/above normal EPO levels, respectively (*p* < 0.0001). The majority of EPO measurements were at the time or within 1 year of diagnosis (67%), with the remainder obtained at referral, during the chronic clinical course. Risk stratification disclosed low, intermediate, and high risk status in 45, 33 and 23% of patients, respectively, with no appreciable differences between EPO-stratified subsets.

**Table 1:** Laboratory and clinical characteristics of 177 untreated patients with World Health Organization-defined chronic phase essential thrombocythemia stratified by serum erythropoietin level

Variables	All ET patients (*n*=177)	ET patients with subnormal EPO (*n*=62)	ET patients with normal or above normal EPO (*n*=115)		*P* value
Age in years; median (range)	51 (18–88)	52 (20–88)	50 (18–87)		0.60
Males; *n* (%)	52 (29)	19 (31)	33 (29)		0.79
Serum EPO levels, mIU/mL; median (range)	5.2 (<1–25)	2.6 (<1–3.9)	7.2 (4–25)		**<** **0.0001**
Timing of serum EPO draw; *n* (%)					
At referral, within 1 year of diagnosis^a^	119 (67)	44 (71)	75 (65)		0.43
Chronic phase, beyond 1 year of diagnosis^a^	58 (33)	18 (29)	40 (35)		
Hemoglobin, g/dL; median (range)	14.3 (12–17.8)^b^	14.7 (12.1–17.8)^b^	13.8 (12–17.1)^b^		**<** **0.0001**
Platelets, ×10^9^/L; median (range) “*N*” evaluable = 176 (99%)	802 (451–2286)	787 (451–2286)	813 (451–1878)		0.31
Platelets >1000 × 10^9^/L; *n* (%) “*N*” evaluable = 176 (99%)	37 (21)	10 (16)	27 (24)		0.23
Leukocytes, ×10^9^/L; median (range) “*N*” evaluable = 176 (99%)	8.6 (3.9–28.1)	9.7 (4–28.1)	8.2 (3.9–22)		**0.0001**
Leukocytes ≥11 × 10^9^/L; *n* (%) “*N*” evaluable = 176 (99%)	36 (20)	19 (31)	17 (15)		**0.02**
LDH at diagnosis, U/L; median (range) “*N*” evaluable = 149 (84%)	198 (100–662)	211 (131–662)	189 (100–358)		0.07
Karyotype “*N*” evaluable = 149 (84%)					
Normal; *n* (%)	141 (95)	52 (93)	89 (96)		0.46
Abnormal; *n* (%)	8 (5)	4 (7)	4 (4)		
Driver mutational status “*N*” evaluable = 147 (83%)					
*JAK2; n* (%)	107 (73)	52 (95)	55 (60)		<**0.0001**
*CALR*; *n* (%)	24 (16)	3 (5)	21 (23)		
*MPL; n* (%)	2 (1)	0 (0)	2 (2)		
Triple-negative*; n* (%)	14 (10)	0 (0)	14 (15)		
*CALR* subtype; *n* (%)					
*CALR* type 1/like	14 (58)	3 (100)	11 (52)		0.06
*CALR* type 2/like	10 (42)	0 (0)	10 (48)		
History of any thrombosis at or prior to diagnosis; *n* (%)	31 (18)	14 (23)	17 (15)		0.2
History of venous thrombosis at or prior to diagnosis; *n* (%)	17 (10)	7 (11)	10 (9)		0.58
History of arterial thrombosis at or prior to diagnosis; *n* (%)	14 (8)	7 (11)	7 (6)		0.23
History of any thrombosis after diagnosis; *n* (%)	21 (12)	11 (18)	10 (9)		0.08
History of venous thrombosis after diagnosis; *n* (%)	10 (6)	4 (6)	6 (5)		0.74
History of arterial thrombosis after diagnosis; *n* (%)	14 (8)	8 (13)	6 (5)		**0.04**
Conventional risk stratification					
Low risk; *n* (%)	79 (45)	26 (42)		53 (46)	0.16
Intermediate risk; *n* (%)	58 (33)	17 (27)		41 (36)	
High risk; *n* (%)	40 (23)	19 (31)		21 (18)	
Fibrotic transformations; *n* (%)	6 (4)	0 (0)		6 (5)	**0.02**
Leukemic transformations; *n* (%)	7 (4)	2 (3)		5 (4)	0.71
Polycythemic transformations; *n* (%)	3 (2)	3 (5)		0 (0)	**0.01**
Follow-up in years; median (range)	6.6 (0–45)	6.7 (0–45)		6.5 (0–25)	0.43
Deaths; *n* (%)	29 (16)	10 (16)		19 (17)	0.95

*WHO* World Health Organization, *ET* essential thrombocythemia, *EPO* erythropoietin, *LDH* lactate dehydrogenase, *JAK2* Janus kinase 2, *CALR* calreticulin, *MPL* myeloproliferative leukemia virus oncogene

^a^The following strict conditions were followed for EPO sample collection: (i) all patients were in chronic phase with no evidence of myelofibrotic or leukemic transformation; (ii) all patients were currently untreated (and those with EPO levels drawn at diagnosis were also previously untreated) with cytoreductive agents; (iii) all patients had hemoglobin levels above sex-adjusted values for anemia; and (iv) patients with hemoglobin values exceeding the threshold for PV criteria had clinical and morphological parameters carefully assessed and all cases of masked PV systematically excluded

^b^Essential thrombocythemia patients presenting with concomitant hemoglobin levels exceeding the threshold for PV but without meeting WHO-defined criteria for PV were found to have either transient elevations in hemoglobin or clear underlying causes of secondary polycythemia such as hypoxia, smoking, or elite athletic training, among others

Bold values identify *P-*values that are significant

ET patients with subnormal EPO levels presented significantly higher hemoglobin (median 14.7 vs 13.8 g/dL, range: 12.1–17.8 vs 12–17.1; *p* < 0.0001) and leukocyte counts (median 9.7 vs 8.2 × 10^9^/L, range: 4–28.1 vs 3.9–22; *p* = 0.0001), as well as greater prevalence of leukocyte counts ≥11 × 10^9^/L (31 vs 15%; *p* = 0.02) and a trend towards higher lactate dehydrogenase (211 vs 189 U/L; *p* = 0.07) compared to those with normal/above normal levels. Conversely, platelet counts and rates of cytogenetic abnormalities (~5%) were similar across the two groups (Table 1). Driver mutational status was nearly exclusively skewed towards *Janus kinase 2* (*JAK2*) mutations in the subnormal EPO cohort (95 vs 60%; *p* < 0.0001) and, interestingly, of those with subnormal EPO harboring calreticulin mutations (*n* = 3), exclusively type 1 variants were represented (100%). Few patients in either group presented palpable splenomegaly at diagnosis, and while both subgroups had a similar history of thrombosis at or prior to diagnosis (18% overall), those with subnormal EPO values had a significantly higher rate of arterial thrombosis after diagnosis (13 vs 5%; *p* = 0.04) and a trend towards heightened overall (venous and arterial) thrombotic risk post diagnosis (18 vs 9%; *p* = 0.08) (Table 1). Rates of leukemic transformation were low and did not differ substantially between the two groups (3 vs 4%; *p* = 0.71); however, importantly, all three of the documented polycythemic transformations (*p* = 0.01) and none of the six myelofibrotic progressions involved those with subnormal circulating EPO concentrations (*p* = 0.02). Median follow-up was 6.7 and 6.5 years with 10 (16%) and 19 (17%) deaths recorded in the subnormal vs normal/above normal serum EPO sub-categories, respectively (Table 1).

**Fig. 1:**
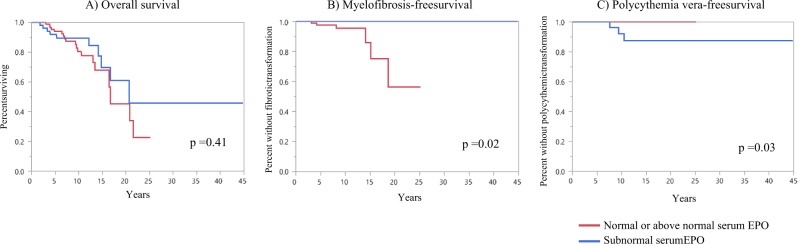
Comparison of overall, myelofibrosis-free, and polycythemia vera-free survival in 177 untreated patients with essential thrombocythemia stratified by serum erythropoietin (EPO) level

Event curves for overall, myelofibrosis-free, and polycythemia vera-free survival are presented in Fig. 1. While overall survival was unaffected by serum EPO levels (*p* = 0.41) (Fig. 1a), levels below the normal reference limit were significantly associated with superior myelofibrosis-free survival (*p* = 0.02)(Fig. 1b) but, conversely, inferior polycythemia vera-free survival (*p* = 0.03) (Fig. 1c).

Serum erythropoietin has long been entrenched in the pathophysiological underpinnings and diagnostic schema of polycythemia vera and, insofar as PV and ET represent related entities in a disease continuum^12,13^, serum EPO has been surprisingly understudied in the latter. Although limitations of the current study included its retrospective nature, unavailability of *JAK2* allele burden data in a sufficient number of informative cases, and preclusion of endogenous erythroid colony and red cell mass testing, which were not feasible, it did endeavor to address previously unexplored, critical questions surrounding subnormal EPO levels in ET—specifically, what are their incidence and clinical significance, if any. Our observations, while requiring further validation, point to the not infrequent finding of subnormal EPO levels in ET and, furthermore, reveal a phenotypic portrait mimicking that of PV: higher hemoglobin levels and leukocyte counts, *JAK2* mutational clustering, and a greater propensity towards arterial thrombosis after diagnosis. Moreover, there appears to be a significant association between subnormal EPO levels and outcomes in ET as patients with suppressed levels may have a greater likelihood of converting to PV but also, interestingly, may exhibit some protection from myelofibrotic transformation, a potentially fatal complication. Whether EPO is driving this disease variability or this simply reflects an epiphenomenon is unclear and requires further study. Regardless, the current observations provide new and meaningful insight into the phenotypic and prognostic specificity annotated by subnormal EPO levels in ET and introduce the possibility of considering serum EPO measurement in the evaluation and risk stratification of patients with ET.
